# Streptococcal Immunity Is Constrained by Lack of Immunological Memory following a Single Episode of Pyoderma

**DOI:** 10.1371/journal.ppat.1006122

**Published:** 2016-12-27

**Authors:** Manisha Pandey, Victoria Ozberk, Ainslie Calcutt, Emma Langshaw, Jessica Powell, Tania Rivera-Hernandez, Mei-Fong Ho, Zachary Philips, Michael R. Batzloff, Michael F. Good

**Affiliations:** 1 Institute for Glycomics, Gold Coast Campus, Griffith University, Brisbane, Queensland, Australia; 2 School of Chemistry and Molecular Biosciences, University of Queensland, Brisbane, Australia; Boston Children's Hospital, UNITED STATES

## Abstract

The immunobiology underlying the slow acquisition of skin immunity to group A streptococci (GAS), is not understood, but attributed to specific virulence factors impeding innate immunity and significant antigenic diversity of the type-specific M-protein, hindering acquired immunity. We used a number of epidemiologically distinct GAS strains to model the development of acquired immunity. We show that infection leads to antibody responses to the serotype-specific determinants on the M-protein and profound protective immunity; however, memory B cells do not develop and immunity is rapidly lost. Furthermore, antibodies do not develop to a conserved M-protein epitope that is able to induce immunity following vaccination. However, if re-infected with the same strain within three weeks, enduring immunity and memory B-cells (MBCs) to type-specific epitopes do develop. Such MBCs can adoptively transfer protection to naïve recipients. Thus, highly protective M-protein-specific MBCs may never develop following a single episode of pyoderma, contributing to the slow acquisition of immunity and to streptococcal endemicity in at-risk populations.

## Author Summary

GAS skin infections pose a significant health problem in the tropics. They are highly prevalent in developing countries as well as amongst the Indigenous populations of developed countries. In at-risk impoverished communities the epidemiology of GAS infections is very dynamic, leading to very high rates of streptococcal-associated serious pathology including rheumatic heart disease, glomerulonephritis and invasive GAS disease. Immunity to GAS takes over 20 years to develop and this has been attributed to sequence diversity of the type-specific surface M-protein. There are more than 250 different strains of GAS and it known that antibodies to the amino-terminal segment of the M-protein can kill organisms in a strain-specific manner *in vitro*. In the present study, using four different strains of GAS isolated from the skin lesions of Aboriginal patients in the Northern Territory of Australia, we make the discovery that skin infection does not induce long-lived type-specific immunity. However, following reinfection with the same strain memory B cells are generated and long-term strain-protective immunity then develops. The dependence on reinfection for the development of strain-specific immunity compounds with antigenic diversity of the M-protein and provides a rational explanation for the very slow acquisition of streptococcal immunity.

## Introduction

The central role of immunity is to prevent re-infection with the same organism. For example, long-lasting immunity prevents re-infections with, measles, smallpox, and pertussis [1–4]. For other organisms that exist as multiple strains (such as influenza virus), infection does not lead to general immunity but induces life-long strain-specific immunity and neutralizing antibody responses [5]. In healthy individuals, an infection or antigen exposure activates B-cells to form germinal centres in secondary lymphoid tissue with T-follicular helper (T_FH_) cells providing factors for growth and differentiation. Within the germinal centre they become antibody-secreting cells (ASCs) and undergo class switching and somatic hypermutation with the affinity of the antibody increasing. After 7–10 days, memory B cells (MBCs) form and circulate to other sites in preparation for future antigenic challenge where they mount a rapid secondary response and long-lived plasma cells (LLPCs) form and migrate to the bone marrow [6]. Immunological memory depends on MBCs but the factors that regulate their formation are neither well known nor understood.

Group A streptococci (GAS) are Gram positive cocci that infect either the naso-pharynx or the skin. It is estimated that in excess of 500,000 lives are lost prematurely each year as a result of GAS infections [7]. The epidemiology of infections is highly dynamic in at-risk impoverished communities, with streptococci of multiple strains sequentially infecting children, such as occurs in many remote Australian Aboriginal communities [8,9]. This results in extreme endemicity and very high rates of streptococcal-associated serious pathology, including rheumatic heart disease [10–12], post streptococcal glomerulonephritis [13,14] and invasive GAS disease [15,16]. Infection does not lead to general streptococcal immunity. This is largely attributed to expression of virulence factors that impede innate immunity (including those inhibiting complement, neutrophil chemotaxis and blood clotting) [17] and the significant sequence diversity of the M-protein, itself a major virulence determinant and target of serotype-specific opsonizing antibodies [18–20]. There are over 150 distinct strains based on the serological M-types [21] and over 250 *emm* types, based on the amino-terminal sequencing of the M-protein gene [22].

Little is known about the induction of strain-specific antibody responses and immunity as a result of streptococcal infection. Reports from the middle of the last century showed the presence of M type-specific antibodies in the blood of children convalescing from GAS pharyngitis [23,24]. Wanamaker et al [25] demonstrated that the presence of type-specific antibodies correlated with a 6-fold reduction in the risk of homologous pharyngeal infection. Lancefield [26] then reported the persistence of strain-specific antibodies in some individuals 4–32 years after a GAS infection; however, other individuals who had a known GAS infection did not demonstrate any type-specific antibodies. There was no correlation between the persistence of these antibodies and the severity of infection. Recently, Bencivenga et al [27] reported that 1 of 2 subjects who had confirmed rheumatic fever 45 years previously had persistent opsonic antibodies against the infecting strain. It was postulated that the higher levels of antibodies in the single individual might have arisen due to additional infections resulting in boosting of antibody levels. To our knowledge this was the first report of persistence of antibodies following streptococcal pharyngitis since the Lancefield study of 1959 but neither study was able to elucidate the factors regulating immunity to pharyngeal infection. There are no other reports on what factors may influence immune induction and persistence.

Even less is known regarding the acquisition and persistence of immunity resulting from streptococcal skin infection (pyoderma). Bisno and Nelson [28] reported that only 2 of 17 children with pyoderma developed type-specific antibodies. In addition, poor anti-streptolysin O and anti-streptococcal NADase responses are observed after skin infection [29]. While pyoderma is prevalent in tropical settings, including amongst Indigenous peoples of Australia, and skin is a common portal of entry for invasive GAS (iGAS) infections [16,30], no studies have investigated factors that regulate induction of immunity to GAS pyoderma in these or other populations.

To ask whether a sub-optimal immune response to streptococcus might be contributing to the high endemicity of streptococcal infections in at-risk communities, we used a mouse model for pyoderma. We sequentially infected mice with the same or with different endemic and reference strains of GAS following which we estimated the streptococcal bio-burdens in skin and blood and correlated these with the serotype-specific antibody responses and with the development of memory B cell responses. The data reveal a novel aspect of GAS immunobiology that if applied to the human situation contributes to the excessively high rates of endemicity and GAS-related pathology in at-risk communities.

## Results

### Streptococcal immunity following—homologous and heterologous infections

**Fig 1:**
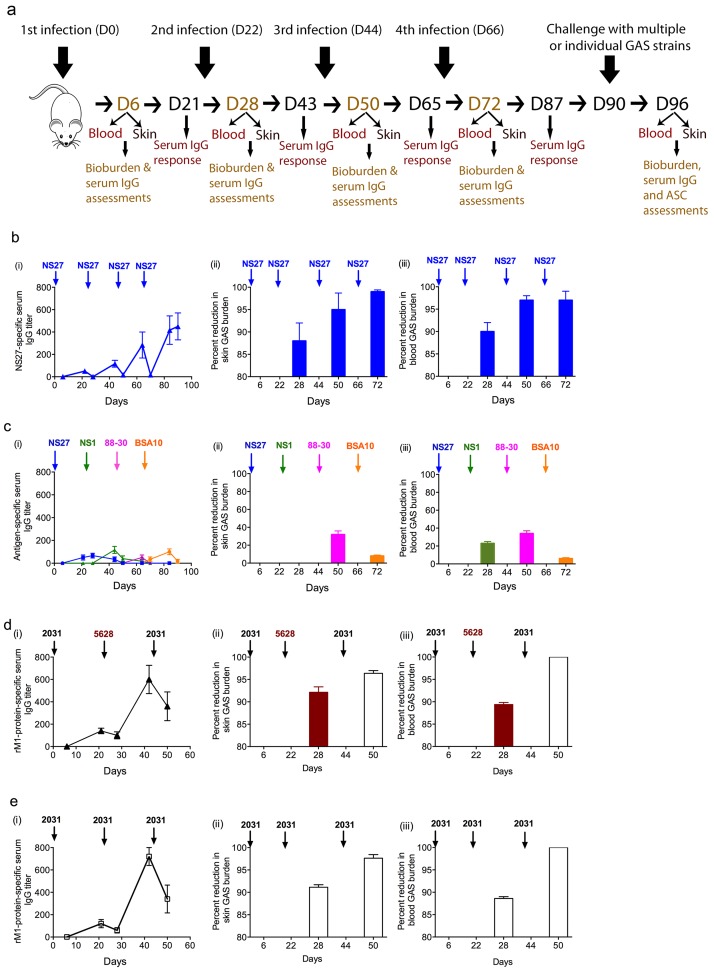
Immunity following sequential GAS infections. BALB/c mice (n = 20/group; female, 4–6 weeks old) were sequentially infected multiple times with (b) NS27 GAS, (c) four different GAS strains NS27, NS1, 88–30 and BSA10, 2 different M1 strains 2031 and 5628 GAS (d) or three times with 2031 GAS (e) via the skin route of challenge. Each infection was 3-weeks apart. On days 6 and 21 post each infection serum sample were collected and M-protein N-terminal-specific IgG titers were measured with ELISA as shown in the schematic (a). This procedure was repeated after each infection and the IgG titers to the N-terminus serotypic peptide/protein of infecting strains at various time-points are shown (b (i), c (i) d (i) and e (i)). On day 6 post each infection, a designated number of mice (n = 5/group) were culled and bacterial burden in skin (b (ii), c (ii) d (ii) and e (ii)) and blood (b (iii), c (iii) d (iii) and e (iii)) were assessed. Naïve BALB/c mice were used as a challenge control at each time-point. Data are mean ± SEM. In b (ii-iii), c (ii-iii), d (ii-iii) and e (ii-iii) the GAS bioburdens (mean CFU) in control mice ranged from 785,260 to 566,550 for skin and 325,870 to 294,225 for the blood.

To assess induction of serotype-specific immunity, naïve BALB/c mice (group A) were repeatedly infected with the NS27 GAS strain (*emm* type 91) via skin scarification [31] and the development of strain-specific antibodies and protective immunity were monitored (Fig 1a). Following a single infection with NS27, low levels of strain-specific IgG were detected (Fig 1b(i)). These were assessed by ELISA using the strain-specific amino-terminal peptide of NS27 as the capture antigen [32]. IgG levels improved (up to 8-fold) following subsequent infections (Fig 1b(i)). We observed that with each subsequent infection the levels of NS27-specific IgG, having risen, were then depleted in the serum, presumably as a result of binding the infecting organism. Within 3 weeks of a single infection, mice developed profound immunity with over 85% reduction in bacterial burden in skin and blood following a second infection (Fig 1b(ii) and 1b(iii)). This level of protection improved even further following subsequent infections and correlated with the increase in NS27-specific IgG titers.

To dissect the development of immunity following infection with different strains, mice were sequentially exposed to GAS strains of different *emm* types (S1 Table) (NS27→NS1→88-30→BSA10) (group B). Each infection was three weeks apart. We determined strain-specific antibodies by ELISA using the relevant amino-terminal peptides for each M-type [32]. Following the initial infection with NS27, there was again a transient appearance of low levels of NS27-specific IgG (Fig 1c(i)). Subsequent infection with NS1 did not boost NS27-specific IgG levels (Fig 1c(i)); similarly, infection with NS27 did not induce immunity to NS1 in skin (Fig 1c(ii)) or blood (Fig 1c(iii)). Similarly, the second infection (NS1) did not induce immunity to the third strain (88–30) and infection with that strain did not induce immunity to BSA10 (Fig 1c(i)–1c(iii). Thus, prior exposure to a given strain provided neither cross-strain-specific antibody nor cross-strain protective immunity, irrespective of the *emm* type of the infecting strains (Fig 1c(i)–1c(iii)).

To determine whether immunity to a given strain was serotype-specific we infected mice with the 2031 strain, which is an M1 serotype, and re-infected them with either same (2031) or a different strain (5628), which is also an M1 serotype. We observed that mice infected with 2031 showed ≥ 90% reduction in bio-burden when subsequently infected with 5628 three weeks later (Fig 1d(ii) and 1d(iii)). The second infection (with either 2031 or 5628) further enhanced immunity to a third infection with 2031 (Fig 1d & 1e). The level of immunity to 5628 was comparable to the immunity observed followed by two sequential 2031 infections (Fig 1e(i)–1e(iii)).

In summary, the data thus show that skin infection with one particular strain does not induce immunity to a different strain unless that strain has the same M-protein as the initial strain. Thus, at least for the strains used in this study, immunity induced following a skin infection and assessed three weeks later is entirely specific to serotypic determinants expressed on the M-protein.

**Fig 2:**
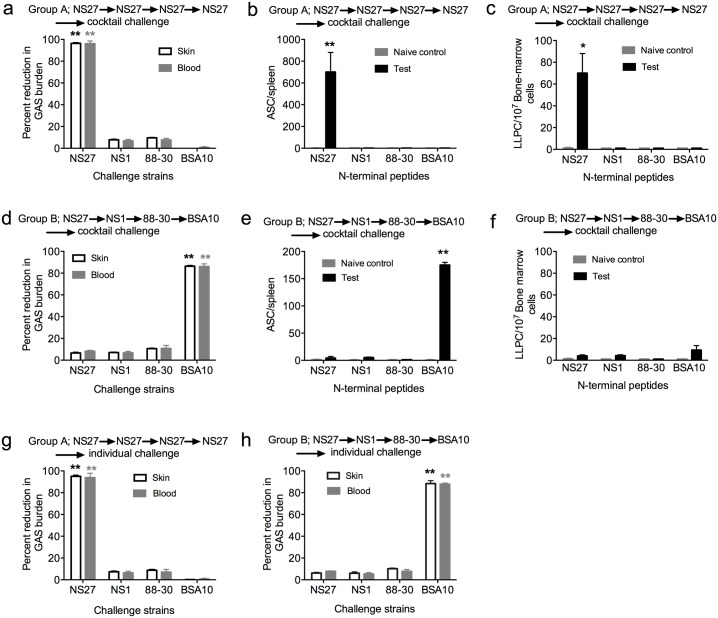
Longevity of immunity following sequential homologous or heterologous infections. To assess the longevity of immunity developed following sequential infections, BALB/c mice (n = 25/group) that had previously received multiple infections with one GAS strain (Groups A (n = 5), C (n = 20) or four different GAS strains (Groups B (n = 5), D (n = 20) were challenged with a cocktail of all 4 GAS strains (figure a and d) or with each individual GAS strain (figure g and h). The cocktail contained all the 4 GAS strains in equal ratio. Naïve BALB/c mice (n = 5) challenged with the GAS cocktail or with each of the 4 strains individually (n = 5 mice/strain), served as controls. The mice were sacrificed on day 6 post-challenge and the percent reduction in bacterial burdens in skin and blood following cocktail challenge (a and d) or individual challenge (g and h) are shown. Reduction in bacterial burden is calculated by taking into account the corresponding naïve control (n = 5/group) and is presented as percent reduction. In a, d, g and h the GAS bioburdens (mean CFU) in control mice ranged from 823,300 to 754,350 for skin and 375,510 to 278,350 for the blood. To assess the longevity of immune responses following sequential infection, a designated number of mice from group A and B were culled at the time of challenge. The spleen and bone marrow were harvested and the number of antibody secreting cells specific for the N-terminal peptide for each infecting GAS strain was enumerated using ELISPOT (group A (b and c) and group B (e and f). Data for each bar are mean ± SEM. Statistical analysis was carried out using one-way ANOVA with Tukey’s post-hoc test to determine significance between the groups for skin* and blood* samples. *p<0.05; **p< 0.01.

We then asked whether immunity to a given strain would be influenced by co-infection with multiple strains of GAS, as is commonly found in streptococcal-endemic areas [9]. To assess this, other mice from the experimental groups (A and B) were then challenged with a cocktail of GAS strains. The cocktail contained, in equal ratio, the four strains: NS27; NS1; 88–30 and BSA10. We assessed strain-specific immunity following challenge by rendering the strains resistant to different antibiotics and then enumerating the number of resistant bacteria growing on antibiotic-laced plates (S1 Table). Following challenge, the group that had received four sequential infections with NS27 demonstrated >95% reduction in the NS27 bacterial burden both in skin and blood in comparison to the naïve control mice (p<0.01) (Fig 2a). There was no reduction in the bacterial loads for the other strains present in the cocktail. The NS27 protection correlated with increases in IgG titers specific for the N-terminal peptide of the NS27 M-protein (Fig 1b), and the numbers of M-protein-specific ASCs in the spleen, and LLPCs in the bone marrow of infected mice at the time of challenge (Fig 2b and 2c). However, following cocktail challenge of group B (which had received four sequential heterologous infections [NS27→NS1→88-30→BSA10]), we observed that mice were protected only against BSA10 with >80% reduction in the bacterial burden both in skin and blood (p<0.01) (Fig 2d). The protection correlated with significant numbers of BSA10-specific ASCs in the spleen (Fig 2e). Very low numbers of LLPCs were detected in bone marrow at this stage (Fig 2f). To exclude the possibility that combining various GAS strains in a cocktail might have affected the virulence of individual strains, the challenge study was repeated with each individual GAS strain. Thus, further mice (groups A and B) exposed to four sequential infections (NS27→NS27→NS27→NS27) or (NS27→NS1→88–30→BSA10) were challenged with each of the four strains individually. The results were consistent with the previous observations, demonstrating protection against NS27 only (group A; Fig 2g) or BSA10 only (group B) (Fig 2h).

To further confirm that this was not an artefact of the experimental design or a strain-specific observation, we repeated the cocktail challenge experiment but changed the order of prior GAS strain infections (NS1→88–30→BSA10; or NS27→NS1→88–30; or NS27→88–30→NS1). In each experimental scenario the mice were protected against only the last of the sequential infections (p<0.01) (S1 Fig). These observations suggested that either the strain-specific primary immune response to GAS was short-lived or that exposure to heterologous strains ablated immunological memory of previous infections**.**

### Number of GAS infections dictates endurance of streptococcal immunity

**Fig 3:**
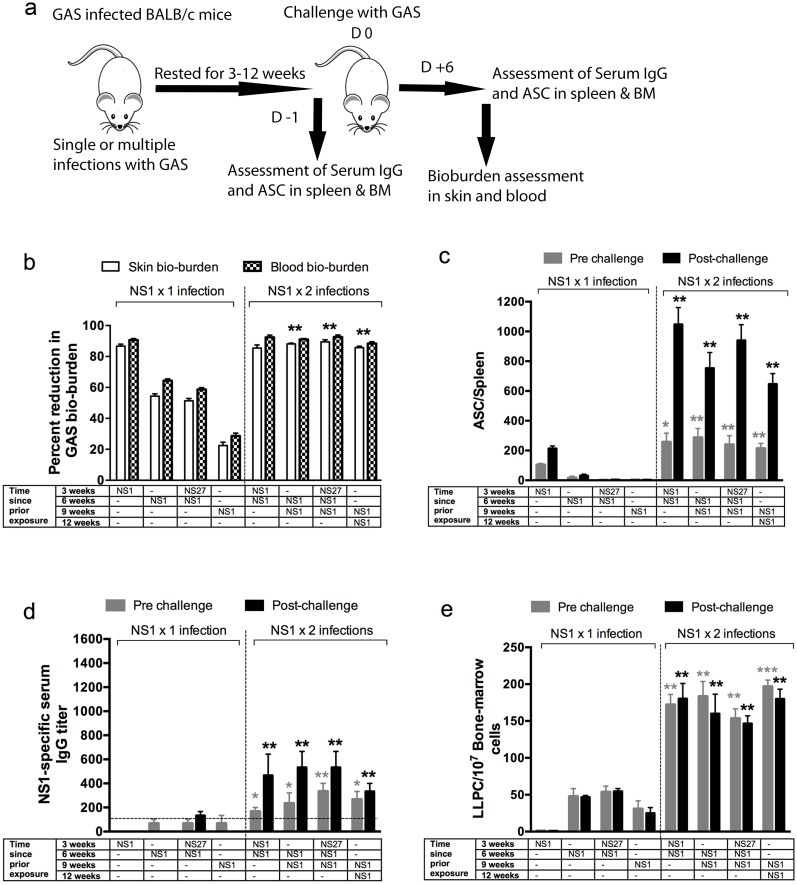
Effect of the number of GAS infections on endurance of protective immunity. To assess if the number of prior homologous and heterologous infections would dictate immunity, 10 mice /group were infected with a representative strains NS1 once or twice and then rested for 3, 6 or 9 weeks. Some cohorts also received an intermittent heterologous infection prior to challenge. As depicted in schematic (a) postrest half of the mice (n = 5/group) were challenged with NS1 GAS and the effect of one or two homologous infections, prior to challenge, on skin and blood bioburdens are shown (b). Age-matched naïve BALB/c mice (n = 5), challenged in parallel, with NS1 GAS were used as controls. GAS bioburdens (mean CFU) in control mice ranged from 845,300 to 797,450 for skin and 242,470 to 212,300 for the blood. Development of serological memory following one or two sequential homologous infections was also followed. To assess NS1-specific IgG-secreting cells, ELISPOT assays were performed. Designated numbers of mice (n = 5/group) from each cohort were culled before or 6 days after the NS1 challenge and NS1-specific ASCs enumerated in their spleens (c). BALB/c mice receiving one or two NS1 infections were assessed for NS1-specific IgG before and after homologous challenge (d). To investigate the long-lived plasma cells, ELISPOT were performed with bone-marrow cells and the numbers of NS1-specific LLPCs are shown (e). Statistical analysis was carried out using ANOVA with Tukey’s post-hoc test to determine significance between the groups (NS1 x 1 versus NS1 x 2 infections with colour of the asterix* denoting the groups being compared). *p<0.05; **p< 0.01, ***p<0.001.

To address this, additional experiments were undertaken with two strains (NS1 and NS27). Mice received a single infection with NS1 before they were challenged with the same strain at varying times up to nine weeks later (Fig 3a). Protective immunity was again assessed in skin and blood. We demonstrated that immunity was dependent on the duration of time between exposure and challenge (Fig 3b). We observed 86% and 90% reduction in skin and blood bacterial burden respectively if mice were challenged within three weeks. However, protection was dramatically reduced (22–55% in skin and 28–64% in blood; p<0.05–0.01) if it was assessed beyond three weeks. We also measured the ability of the challenge infection to boost the IgG titers and the numbers of ASCs in the spleen and bone marrow. We observed that in mice which had a single infection 3, 6, or 9 weeks previously, a subsequent infection did not boost either ASC levels in the spleen nor serum antibody responses, suggesting that MBCs had not formed (Fig 3c and 3d). The low level of protection that was observed (e.g. ~25% protection at 9 weeks post infection) can be explained by the presence of low numbers of antigen-specific LLPCs in the bone marrow (Fig 3e).

However, we observed that re-infection with the same strain did induce enduring strain-specific immunity to a further infection with 84–89% and 87–95% reduction in skin and blood bio-burden respectively persisting for more than 12 weeks following the initial infection, irrespective of whether or not there was an intervening infection with a different strain (Fig 3b). Two sequential infections with NS1 (three weeks apart) significantly increased the numbers of splenic ASCs and their response to a further infection (p<0.05–0.01), and improved the NS1-specific IgG titers as well as the IgG response to a further infection (p<0.05–0.01), compared to mice that had received a single infection (Fig 3c and 3d). The numbers of LLPCs in the bone marrow increased significantly as well (Fig 3e). Similar observations were made when the experiments were conducted with NS27 GAS (S2 Table). The data were consistent with the induction of MBCs following two infections that were then responsible for the boost in ASC numbers and the IgG titer following a subsequent infection.

To confirm that these observations were not specific to the particular mouse strain used in this study, a set of identical experiments was carried out in outbred SWISS mice. We observed that similar to BALB/C mice, development of protective immunity in SWISS mice also required two sequential infections within 3 weeks (S2a Fig). These mice also demonstrated the necessity for two sequential infections to develop enduring strain specific IgG and ASC responses (S2b–S2d Fig). The data from both mouse strains were consistent with the need for two infections with the same strain to induce MBCs. Although a rapid antibody response following infection is a hallmark of MBCs (Figs 3d and S2C), existing antibody from LLPCs can make the interpretation more difficult.

### Re-infection is required for induction of B-cell memory

**Fig 4:**
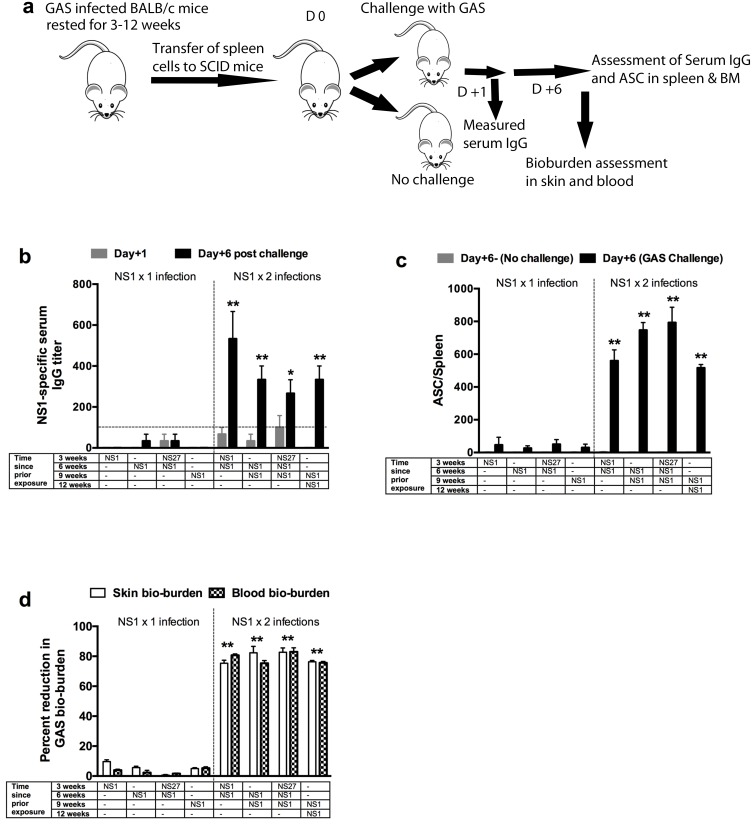
Role of antigen specific memory B-cells in immunity. To investigate the development of memory B cells following sequential GAS exposures, two GAS strains NS1 and NS27 were used. Mice received single or multiple infections with either or both strains and rested for 3, 6 or 9 weeks before they were culled and their spleens harvested. As depicted in the schematic (a) the splenocytes from BALB/c mice (n = 15/group) that received single or multiple GAS infections were either utilised to assess NS1-specific ASC responses by ELISPOT (n = 5) or transferred into naïve SCID (n = 10) mice. Half of the recipient SCID mice (n = 5) were challenged with NS1 GAS. The mice (n = 5) that received primed splenocytes but did not receive a GAS challenge were used as controls. NS1-specific IgG levels before and 6 days after homologous challenge were measured (b). The dotted line represents the base antibody levels. To assess NS1-specific antibody-secreting cells, ELISPOT assays were performed. Designated numbers of SCID mice from each cohort (with or without NS1 challenge) were culled 6 days after adoptive transfer and NS1-specific ASCs enumerated in their spleens (c). The mice that received primed splenocytes but did not receive a GAS challenge were used as controls. To investigate the functionality of memory B-cells, SCID mice were challenged with NS1 GAS. Percent reduction in skin and blood bacterial burden in comparison to naïve challenge control (n = 5/group) was calculated and is shown as mean ± SEM (c). The GAS bioburdens (mean CFU) in control mice ranged from 279,350 to 225,400 for skin and 215,650 to 185,320 for the blood. Statistical analysis was performed using one-way ANOVA with Tukey’s post-hoc test to determine significance between the groups (NS1 x 1 versus NS1 x 2 infections with colour of the asterix* denoting the groups being compared)). *p<0.05; **p< 0.01.

To identify MBCs more accurately, spleen cells from previously infected BALB/c and control mice were adoptively transferred into naïve immunodeficient SCID mice. This protocol does not transfer LLPCs, which are resident in bone marrow, and as such the background effect of existing antibody is removed. Post transfer, serum antibodies and ASCs specific for the serotype of the infecting strain were analysed after one day (background) and again after six days (to measure the ‘boost’ indicative of an MBC response) (Fig 4a). Antigen-specific IgG responses in recipient mice following single NS1 infections of donor mice 3, 6, or 9 weeks previously were extremely low at both days one and six (Fig 4b). Thus, there were very few ASCs in the donor cells that were transferred and no MBCs. However, two sequential infections of donor mice with NS1 three weeks apart significantly improved the NS1-specific IgG boost response in the recipients at day six-post challenge (p<0.05–0.01) (Fig 4b). Similarly, following two infections of donor mice with NS1, recipient mice demonstrated a significant boost in the numbers of ASCs in their spleens compared to recipients of spleen cells from donor mice with a single infection (≥ 600 *vs* <50) (p<0.01) (Fig 4c). MBCs persisted at unabated numbers for at least nine weeks after the second infection (Fig 4c). Memory was not ablated by a heterologous infection. It should be noted that when the donor mice were re-infected after 3 weeks, the initial skin lesion had completely healed and there was no residual streptococcal bio-burden.

To assess whether the recipient SCID mice were protected, we euthanized mice from each group on day six post-challenge and assessed protection in skin and blood by enumerating total colonies. We observed only limited protection (<12% and <5% reduction in skin and blood burden respectively) in recipients of spleen cells from mice that had a single infection (Figs 4d and S3). Nevertheless, we observed that spleen cells from donor mice that had been infected twice, three weeks apart (3 and 6 weeks; 6 and 9 weeks; or 9 and 12 weeks prior to transfer), were highly effective in protecting recipient mice with ~80% reduction in bacterial burden in skin and blood (Figs 4d and S3). It was noteworthy that the MBCs protected the SCID mice even though these mice did not have detectable antibodies at the time of challenge.

**Fig 5:**
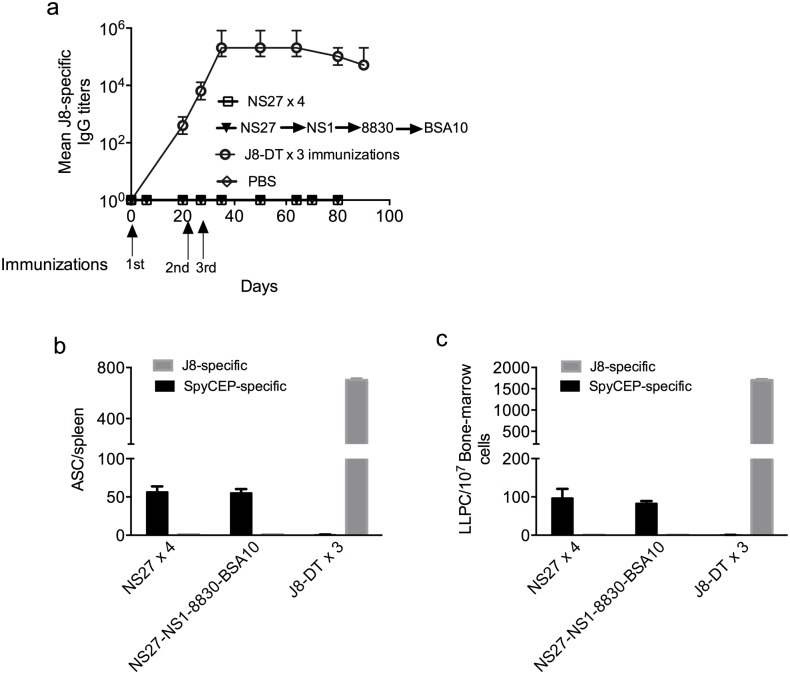
Effect of sequential infections on development of immunity to conserved epitopes of GAS. The development of J8-specific antibodies in mice (n = 5/group) that were sequentially infected with homologous (NS27x4) or heterologous GAS (NS27, NS1, 88–30 and BSA10) was followed. Mice (n = 5) vaccinated with J8-DT were used as positive control. On days 6 and 21 post each infection, serum sample were collected and J8-specific IgG titers at various time-points are shown (a). On day 90 (3-weeks post last infection), mice were culled and spleen and bone marrow harvested. J8 and SpyCEP-specific antibody secreting cells in spleen (b) and bone marrow (c) were enumerated and are shown. Data are mean ± SEM.

We were curious if sequential infections would generate immunity to the conserved epitopes of GAS. A conserved peptide epitope in the C3 repeat region of the M-protein, (J8), is able to induce strain-transcending immunity to skin infection [31,33]. We thus asked whether the level of immunity following infection was related to the level of J8-specific antibodies in these mice. However, we observed that anti-J8 antibodies were not induced following single or multiple infections with the same strain or with different strains (Fig 5a). Active infection can deplete antibodies from the serum, rendering interpretation of antibody data difficult during an infection [34]; however, the lack of an anti-J8 antibody response was mirrored by a complete lack of induction of anti-J8 ASCs in the spleen and LLPCs in the bone marrow (Fig 5b and 5c). These data are consistent with the human experience where many years of high-level endemic exposure to GAS are required to generate even a low-level antibody response to this conserved epitope [35]. Interestingly, mice receiving sequential infections did generate antibody [33] and ASC responses to the IL-8 protease and streptococcal inhibitor of neutrophil chemotaxis, SpyCEP (Fig 5b and 5c). Again, these data are in line with previous observations where anti-SpyCEP antibodies have been reported in IVIG [36] as well as in convalescent human plasma and, depending on titer, may have a role in immunity [33].

## Discussion

Here, we demonstrate that skin infection with streptococci leads to profound, but short-term, strain- and serotype-specific protection. Infection does not lead to the generation of MBCs that protect against either homologous or heterologous strains. However, two homologous infections within a 3-week period, do establish specific MBC responses that are enduring and protective. We are not aware of previous examples in any system where two infections are required to induce durable strain-specific immunity. We used mice with superficial skin scarification to model the development of immunity in humans, where streptococcus is known to enter only broken skin. To mimic the situation in GAS-endemic areas, where individual skin lesions are often co-infected with multiple strains of GAS simultaneously [9], mice were sequentially exposed to single or multiple GAS strains or were co-infected with multiple strains simultaneously. We found that immunity following a single infection was effective but short-lived.

Four significant reports suggest the observations described here will translate into the human experience. Firstly there is the report of children not developing type-specific antibodies following known streptococcal infections [37] along with the report that antibodies from streptococcal pharyngitis, once induced, are long-lived [26]. Secondly, there is the report that only 12% of children with pyoderma develop antibodies to the infecting strain [28]. Then there is the detailed epidemiological study of McDonald et al [8] demonstrating that different strains of GAS move sequentially through at-risk Aboriginal communities in Australia, although more than one strain exists at one time and each strain persists for at least 6 months. While not studied specifically, this persistence suggests that children within the community are very slow to develop immunity, possibly due to the requirement for re-infection. An early study following GAS infections in early childhood reported the occurrence of new versus reinfections as 25 versus 50 per 100 per annum respectively, throughout the study period of 4 years [38].

Adoptive transfer studies showed that MBCs, after a second infection, were effective in controlling an infection even in the absence of circulating type-specific antibodies and LLPCs at the time of challenge. Spleen cells from singly infected mice could transfer only very limited protection to recipient mice from three to nine weeks post-infection (<10%) compared to spleen cells from donors that had been infected twice (~80%; Fig 4d). There was no evidence that spleen cells from mice singly-infected 3, 6, or 9 weeks prior to adoptive transfer and challenge contained MBCs as shown by the lack of any boosting in either these mice (Fig 3) or in SCID mice that had received spleen cells from them (Fig 4) following a challenge infection. In contrast, cells from doubly infected mice were shown to contain MBCs by their rapid response (within six days) with ASCs and the rapid production of antibody following challenge. Immunity induced by repeated infections with a particular strain, and mediated by MBCs, was strain-specific. An intermediate infection with a different strain did not lessen the degree of immunity to the doubly infecting strain. These data might in part explain the situation in the Aboriginal communities of northern Australia where the highest rates of acute rheumatic fever in the world have been reported [39]. The high prevalence of pyoderma (≥70%) among children throughout the year with up to 14 genetically different GAS strains circulating at any one time [40] suggests a lack of natural immunity in these populations, consistent with our observations here.

We did not determine the specificity of the MBCs mediating protection; however, following adoptive transfer into SCID mice and re-infection there was an anamnestic response of antibodies to the amino-terminal epitopes of the M-protein (Fig 4b). We previously showed that vaccination with these epitopes induces highly effective bactericidal antibodies [32,41]. Infection with one strain did not induce immunity to a different strain with a different *emm* type; however infection with one strain did induce immunity to a different strain but with the same *emm* type. However, repeated infections with the same or different strains of GAS did lead to a broadening of the antibody repertoire as shown by the induction of antibodies to the IL-8 protease, SpyCEP [33]; and, depending on titer, these antibodies are known to play a role in protection [31,36]. Thus, it is likely that the protective MBCs would have multiple specificities, of which the amino-terminal region of the M-protein was dominant and most critical. However, one epitope that did not stimulate antibody production or MBCs in re-infected mice was the conserved vaccine candidate epitope of the M-protein, J8, demonstrating that this epitope is entirely cryptic (Fig 5a–5c).

We do not know why MBCs are not induced after a streptococcal infection. However, isotype switching has occurred by three weeks and the antibodies are strongly protective, suggesting that germinal centres, from which MBCs originate, have formed. Hyper immune stimulation may explain our observations. Chronic infections with HIV or malaria parasites are known to expand populations of exhausted or atypical MBCs [42,43], which are hypo-responsive to *in vitro* stimulation. Atypical MBCs have not been described for streptococcus, but it is possible that bacteraemia and antigen load have led to exhaustion of MBCs. Our observations that bacterial loads are much reduced during a second infection, where MBCs are formed, are consistent with that hypothesis. However, the experiences with smallpox and influenza argue against this. Smallpox is known to induce life-long immunity [3] and neutralizing antibodies persisted for life following the 1918 Spanish influenza epidemic [5]. In both these infections there was a very high antigenic load. Streptococcus is known to express numerous immune-modulating factors [17] and it is possible that these have directly impeded the T_FH_ cells and/or the B cells within the germinal centre, blocking MBC development.

GAS is human specific pathogen and therefore to study its pathogenesis an appropriate mouse model as well as animal-adapted GAS strains are critical. The pathology of the murine superficial skin challenge model used here resembles the histopathology of human pyoderma and has been instrumental in understanding pathogenesis and immunity following vaccination ([31,33]. However, while serial passaging in mice enhanced the virulence of the strains used in this study, the role of plasminogen in the pathogenesis of GAS infection remains an important issue for all mouse experiments. GAS streptokinase activates human plasminogen, but not mouse plasminogen, enabling GAS to clear fibrin clots in the skin tissue and more readily enter the blood [44]. While this represents a limitation to the use of mouse models in the study of streptococcal immunity in general, the plasminogen system is only one of many factors exploited by GAS in invasion [45]. Possibly once GAS enters the blood this becomes less relevant. In our study GAS do enter the blood, thus demonstrating virulence in the absence of human plasminogen. It seems unlikely that this would explain the lack of induction of acquired immunological memory.

A further limitation of mouse models in the study of streptococcal immunity is the lack of response of mice to GAS super-antigens. These play a central role in the pathogenesis of toxic shock syndrome in humans. However, toxic shock syndrome leads to immune ablation in humans and the lack of this syndrome in mice could not explain why mice do not develop immunity to GAS infection after a single infection.

Thus, the data presented here paint a picture of streptococcal pathogenesis and endemicity that is more complex than previously thought. The antigenic diversity of the M-protein at its amino terminus was thought to be primarily responsible for immune evasion and virulence factors were known to interfere with innate immunity. While these are undoubtedly major impediments to the development of immunity in children, our data suggest that a child must be exposed on more than one occasion to the same strain for immunological memory to develop to that strain. We had previously hypothesized that antibodies to the conserved J8 epitope of the M-protein would contribute to natural immunity and with heavy persistent infection, most children do eventually develop antibodies to this region of the M-protein [35]; however, this acquisition is very slow and consistent with the data here that four sequential skin infections do not lead to the development of these antibodies. Thus it would appear that, along with antigenic diversity, the requirement for more than one infection with each serotype in order to induce memory represent the most important obstacles to developing acquired immunity to streptococci in early childhood. With repeated exposure over many years, antibodies to conserved antigens including SpyCEP [46], C5a peptidase [47], group carbohydrates [48] and the cryptic conserved epitopes of the M-protein [35] may develop and play significant roles in protection, which then becomes life-long.

## Materials and Methods

### Ethics statement/ Animals

All animal studies were reviewed and approved (AEC protocol # Gly/07/14) by Griffith University’s Animal Ethics Committee in accordance with the National Health and Medical Research Council (NHMRC) of Australia guidelines. Specific pathogen free 4–6 week old female BALB/c, SWISS or SCID mice were sourced from the Animal Resource Centre (Perth, Western Australia).

### Peptides

The peptide J8 (QAEDKVKQSREAKKQVEKALKQLEDKVQ) was synthesized and conjugated to diphtheria toxoid (DT) as described previously [33]. The gene encoding M1 protein (amino acids 13–455) was cloned into pGEX-2T (GE Healthcare Life Sciences), incorporating a carboxy-terminal 6 x His-tag [49]. The amino terminus serotypic peptides for each GAS strain NS27_1-19_ (ADDHPGAVAARNDVLSGFSC), NS1_1-19_ (RVTTRSQAQDAAGLKEKADC), 88–30_1–20_ (DNGKAIYERARERALQELGPC) and BSA10_1-19_ (NSKTPAPAPAVPVKKEATKC) were as previously described [32] and were synthesized at Toth laboratory, University of Queensland (Brisbane) or at China Peptides Co., LTD (Jiangsu, China). All peptides were stored lyophilized or in solution at -20°C.

### Bacterial strains and generation of antibiotic resistance

GAS isolates NS1, NS27, 88-30, BSA10, 2031 were originally obtained from Menzies school of Health Research, (Darwin, NT, Australia). The animal adapted derivative of GAS isolate 5628R was obtained from the Walker laboratory (University of Queensland) [50]. Details of each strain are given in (S1 Table). All the isolates were passaged in mice. To allow for their selection during co-infection experiments, each strain was made resistant to a specific antibiotic by continually replating them with increasing concentrations of antibiotic on blood agar plates (S1 Table).

### Sequential GAS infections and challenge

Naïve BALB/c (inbred) or SWISS (outbred) mice were infected with GAS via the skin route of infection as previously described [31]. To prepare challenge inocula, the GAS strains were grown in Todd-Hewitt broth (THB; Oxoid, Australia) supplemented with 1% (wt/vol) neopeptone (Difco) and in the presence of a specific antibiotic for each strain. The fitness of various GAS strains used in the cocktail was confirmed *in vitro* prior to *in vivo* challenge studies. For CFU enumeration, 10-fold serial dilutions of bacterial cultures were plated in replicates on blood agar plates consisting of the medium described above with 2% agar and 2% horse blood. The broth culture inoculum was adjusted to obtain the intended challenge dose. For sequential infections mice were given 1x10^6^ CFU/mouse. Each sequential infection was followed for up to 3-weeks to confirm the clearance of bacteria from skin and blood prior to subsequent infection. Following sequential homologous (NS27x4) or heterologous (NS27-NS1-8830-BSA10) infections, mice were challenged with a cocktail of all the 4 GAS strains or each strain individually. For challenge experiments with individual strains, the cohorts of sequentially infected mice (homologous or heterologous) were divided into 4 groups and each group was challenged with one of the 4 challenge strains. To confirm serotype-specific immunity, in separate experiments, two distinct GAS strains 2031 and 5628 from the same emm serotype (emm1) were used. For memory experiments two selected GAS strains NS1 and NS27 were used. In all experiments, age-matched naïve mice were used as challenge controls.

### Sample collection and CFU determination

Following each sequential infection, serum and tissue samples were collected at designated time points (Fig 1a). On day 6 post each infection, a designated number (n = 5) mice were euthanized to obtain skin and blood samples for CFU quantification. To allow for detection of current and previous GAS infections, specific antibiotic-laced blood agar plates were used through out the experiments. Similar procedures were employed following final GAS challenges. Six days post cocktail or individual challenge, designated numbers of mice were euthanized and skin and blood samples collected for bio-burden assessment. Where specified, for selected experiments, spleen and bone marrow were also harvested at the same time-point to assess the memory responses.

### Detection of murine antibodies

ELISA was used for the measurement of antigen-specific IgG titers in serum, as described elsewhere [34]. Titertek PVC microplates (MP biomedicals) plates were coated with J8 or N-terminal serotypic peptide. Serum samples were assessed using 2-fold dilutions of 1:100 dilution of serum. Antigen-specific mouse antibodies were detected with HRP-conjugated goat anti-mouse IgG antibody (Bio-rad Laboratories). SIGMA*FAST* OPD (Sigma-Aldrich) was employed as a HRP substrate and absorbance was measured at 450 nm. Antibody titers were defined as the highest dilution that provided an optical density reading at 450nm of > 3SDs above the mean optical density of control wells containing normal mouse serum.

### Detection of antibody secreting cells with ELISPOT

To quantify the number and location of antibody secreting cells (ASCs), bone marrow and splenocytes of naïve or vaccinated-GAS infected and control mice were analysed at specific time-points by ELISPOT. Multiscreen-HA plates were coated with 5 μg/mL of J8 or N-terminal serotypic peptides (NS27, NS1, 88–30 or BSA10) in an alkaline carbonate coating buffer, overnight at 4°C. Isolated spleen and bone-marrow cells were directly tested for IgG-secreting ASCs on these coated plates using published methods [51,52]. The use of J8 or N-terminal serotypic peptides allowed measurement of specific ASCs.

### Adoptive transfer experiments

To investigate memory responses following single or multiple homologous and heterologous infections, splenocytes from GAS infected BALB/c mice were adoptively transferred into naïve SCID mice. Spleens were mashed and passed through a 0.70 μm cell strainer to obtain single cell suspensions. RBC lysis was carried out using ACK lysis buffer (0.15 M NH_4_Cl, 10 mM KHCO_3_ and 0.1 mM Na_2_EDTA; pH 7.4). Following two washes, the cells were counted, resuspended in 200 μL sterile PBS and transferred intravenously into SCID mice. Each mouse received one spleen equivalent.

### Vaccination protocol

Mice were immunized s.c. at the tail base on days 0, 21 and 28 with 30 μg of J8-DT adjuvanted with alum (Alhydrogel [2%]; Brenntag) at a 1:1 ratio (50 μl immunization dose)/mouse. Serum samples were taken prior to and one week after each immunization. Vaccinated mice were also followed for longevity of J8-specific IgG responses until day 90. At specific time-points designated numbers of mice were culled, spleen and bone marrow harvested and ASCs were enumerated.

### Statistical analysis

Data were analysed using GraphPad PRISM version 6.00 for Macintosh. Except where noted, data shown are mean ± Standard Error of Mean (SEM). Statistical differences between two groups were determined using the non-parametric *U* test with p<0.05 considered to be statistically significant. ANOVA with a Tukey’s post-hoc method for multiple comparisons was used for pairwise comparisons. The p values *p*<0.05 was considered to be statistically significant.

## Supporting Information

S1 Table GAS strains used to study immunity following infection with multiple strains. (DOCX)

S2 Table Protection following single or multiple infections with NS27 GAS. (DOCX)

S1 Fig Effect of order of GAS infection on development of immunity. To assess if the order of GAS infection would have an effect on immunity, three cohorts of mice (n = 10) received sequential GAS infections in different orders. In each case, the last infection was either BSA10 (a), 88–30 (b) or NS1 (c). Three weeks post last infection; the mice were challenged with a GAS cocktail as described above. On day 6 post-infection, mice were culled and bacterial burden in skin and blood assessed. Reduction in bacterial burden was calculated taking into account the corresponding naïve control and is presented as percent reduction. Data for each bar are mean ± SEM. Statistical analysis was carried out using one-way ANOVA with Tukey’s post-hoc test to determine significance between the groups. **p< 0.01.
(TIFF)

S2 Fig Effect of the number and length of prior GAS infections on endurance of protective immunity in SWISS mice. To assess if the number and timing of prior GAS infections would dictate immunity, SWISS mice (female 4–6 weeks, n = 15/group) were sequentially exposed once or twice to NS1 GAS and then rested for 3, 6 or 9 weeks. Some cohorts also received an intermittent heterologous infection 3-weeks prior to challenge. As depicted in schematic (Fig 3a) post rest a designated number of the mice (n = 10/group) were challenged with NS1 GAS via the skin route of infection. The effects of one or two prior homologous infections on skin (CFU/skin lesion) and blood (CFU/mL) bioburdens are shown (a). Age-matched naïve SWISS mice (n = 10), challenged in parallel with NS1 GAS were used as controls. GAS bioburdens (mean CFU) in control mice ranged from 953,500 to 435,450 for skin and 682,470 to 415,300 for the blood.
**Development of serological memory following one or two sequential homologous infections.** To assess NS1-specific IgG-secreting cells in the spleen of SWISS mice, ELISPOT assays were performed. Designated numbers of mice (n = 5/group) from each cohort were culled before or 6 days after the NS1 challenge and NS1-specific ASCs were enumerated in their spleens (b). NS1-specific serum IgG titers were also measured before or 6 days post NS1 challenge (c). To investigate the long-lived plasma cells, ELISPOT were performed with bone-marrow cells and the numbers of NS1-specific LLPCs are shown (d). Statistical analysis was carried out using ANOVA with Tukey’s post-hoc test to determine significance between the groups (NS1 x 1 versus NS1 x 2 infections). *p<0.05; **p< 0.01, ***p<0.001.
(TIFF)

S3 Fig Gross pathology at the skin site of infection following adoptive transfer and challenge of SCID mice. BALB/c mice received single or multiple infections with NS1 or both NS1 and NS27 strains and rested for 3, 6 or 9 weeks before they were culled and their spleens harvested. The splenocytes from BALB/c mice that received single or multiple GAS infections were transferred into naïve SCID mice (n = 10/group). To assess the functionality of memory B cells, SCID mice were challenged with NS1 GAS. Six days post challenge mice were euthanized and bioburden assessed. The gross pathology, of representative SCID mice that received splenocytes from singly infected (a) or doubly infected (b) BALB/c mice, following skin challenge is shown. Magnified images of skin lesion (marked with a circle) in some representative mice from various groups are also shown (c).
(TIFF)
