# Supplementary material for: Streptococcal Immunity Is Constrained by Lack of Immunological Memory following a Single Episode of Pyoderma
Source: PLoS Pathog. 2016 Dec 27;12(12):e1006122. doi: 10.1371/journal.ppat.1006122 (PMC5222516; doi:10.1371/journal.ppat.1006122)
Supplement: S1 Table — (DOCX) [file ppat.1006122.s001.docx]

**S1 Table: Strains used to study immunity following infection with multiple strains**

| **GAS strain** | **Selection marker (antibiotic)** | **Isolate origin** | ***emm*-type** |
| --- | --- | --- | --- |
| NS27 | Streptomycin  (200 μg/mL) | Skin | *emm*91 |
| NS1 | Gentamycin  (20 μg/mL) | Skin | *emm*100 |
| 88/30 | Neomycin  (200 μg/mL) | Skin | *emm*97 |
| BSA10 | Gentamycin  (100 μg/mL) | Skin | *emm*124 |
| 2031 | Streptomycin  (200 μg/mL) | Throat | *emm1* |
| 5628R | - | blood | *emm1* |
