# Supplementary material for: Streptococcal Immunity Is Constrained by Lack of Immunological Memory following a Single Episode of Pyoderma
Source: PLoS Pathog. 2016 Dec 27;12(12):e1006122. doi: 10.1371/journal.ppat.1006122 (PMC5222516; doi:10.1371/journal.ppat.1006122)
Supplement: S2 Table — (DOCX) [file ppat.1006122.s002.docx]

**S2 Table: Protection following single or multiple infections with NS27 GAS**

Percent reduction is in comparison with the corresponding naïve control cohorts. GAS bioburdens (mean CFU) in control mice ranged from 785,300 to 747,450 for skin and 312,440 to 252,320 for the blood.
