# Supplementary figures and images for: Streptococcal Immunity Is Constrained by Lack of Immunological Memory following a Single Episode of Pyoderma
Source: PLoS Pathog. 2016 Dec 27;12(12):e1006122. doi: 10.1371/journal.ppat.1006122 (PMC5222516; doi:10.1371/journal.ppat.1006122)

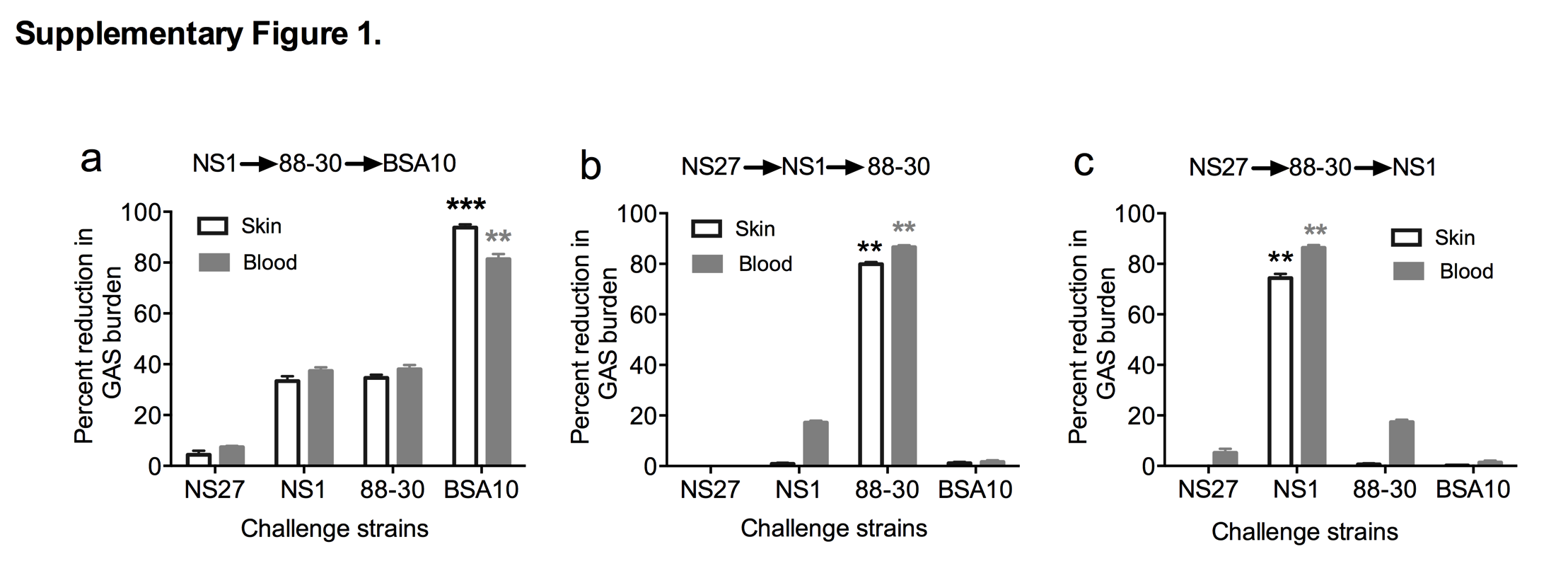

Supplement: S1 Fig — To assess if the order of GAS infection would have an effect on immunity, three cohorts of mice (n = 10) received sequential GAS infections in different orders. In each case, the last infection was either BSA10 (a), 88–30 (b) or NS1 (c). Three weeks post last infection; the mice were challenged with a GAS cocktail as described above. On day 6 post-infection, mice were culled and bacterial burden in skin and blood assessed. Reduction in bacterial burden was calculated taking into account the corresponding naïve control and is presented as percent reduction. Data for each bar are mean ± SEM. Statistical analysis was carried out using one-way ANOVA with Tukey’s post-hoc test to determine significance between the groups. **p< 0.01. (TIFF) [file ppat.1006122.s003.tiff]

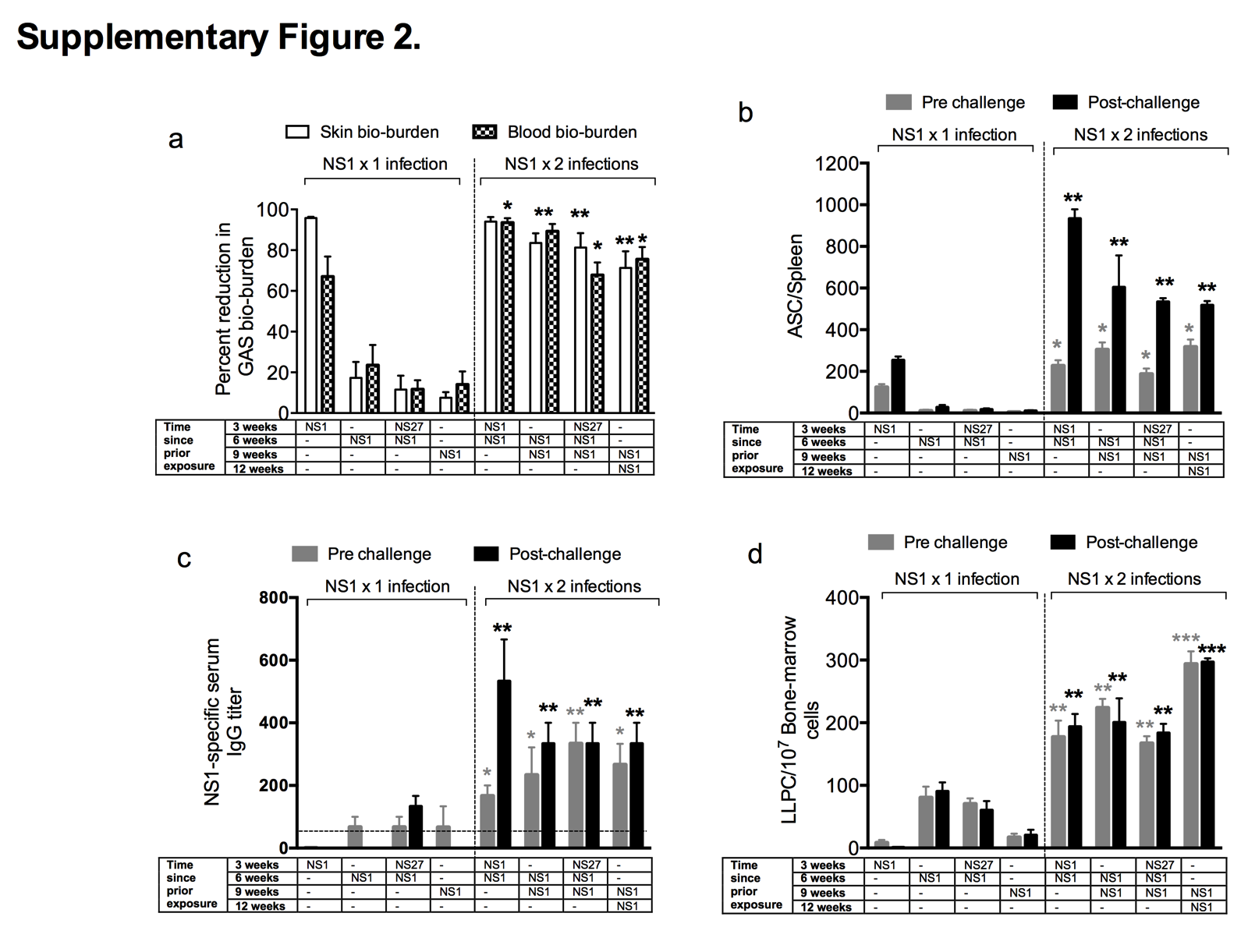

Supplement: S2 Fig — To assess if the number and timing of prior GAS infections would dictate immunity, SWISS mice (female 4–6 weeks, n = 15/group) were sequentially exposed once or twice to NS1 GAS and then rested for 3, 6 or 9 weeks. Some cohorts also received an intermittent heterologous infection 3-weeks prior to challenge. As depicted in schematic (Fig 3a) post rest a designated number of the mice (n = 10/group) were challenged with NS1 GAS via the skin route of infection. The effects of one or two prior homologous infections on skin (CFU/skin lesion) and blood (CFU/mL) bioburdens are shown (a). Age-matched naïve SWISS mice (n = 10), challenged in parallel with NS1 GAS were used as controls. GAS bioburdens (mean CFU) in control mice ranged from 953,500 to 435,450 for skin and 682,470 to 415,300 for the blood. Development of serological memory following one or two sequential homologous infections. To assess NS1-specific IgG-secreting cells in the spleen of SWISS mice, ELISPOT assays were performed. Designated numbers of mice (n = 5/group) from each cohort were culled before or 6 days after the NS1 challenge and NS1-specific ASCs were enumerated in their spleens (b). NS1-specific serum IgG titers were also measured before or 6 days post NS1 challenge (c). To investigate the long-lived plasma cells, ELISPOT were performed with bone-marrow cells and the numbers of NS1-specific LLPCs are shown (d). Statistical analysis was carried out using ANOVA with Tukey’s post-hoc test to determine significance between the groups (NS1 x 1 versus NS1 x 2 infections). *p<0.05; **p< 0.01, ***p<0.001. (TIFF) [file ppat.1006122.s004.tiff]

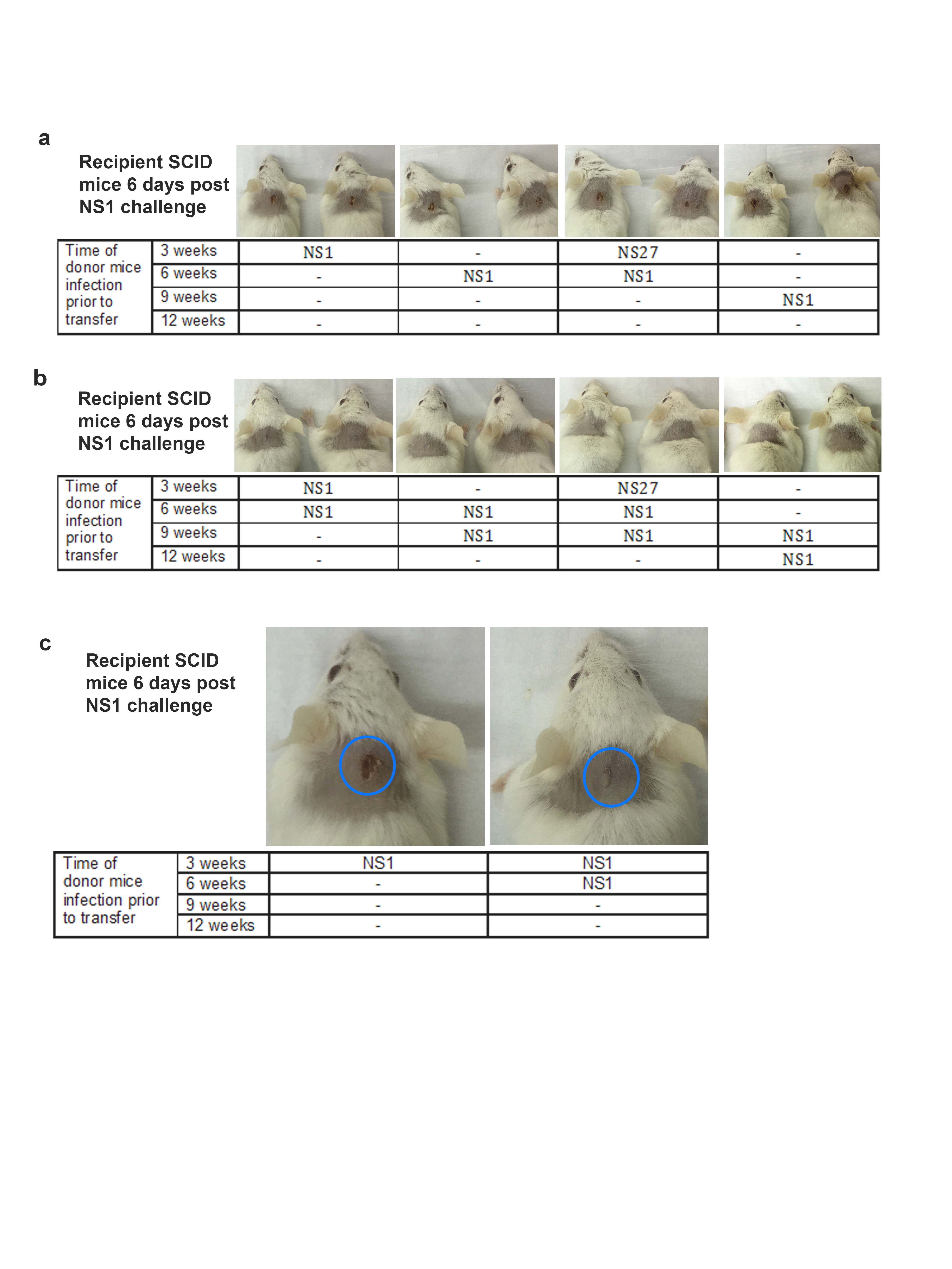

Supplement: S3 Fig — BALB/c mice received single or multiple infections with NS1 or both NS1 and NS27 strains and rested for 3, 6 or 9 weeks before they were culled and their spleens harvested. The splenocytes from BALB/c mice that received single or multiple GAS infections were transferred into naïve SCID mice (n = 10/group). To assess the functionality of memory B cells, SCID mice were challenged with NS1 GAS. Six days post challenge mice were euthanized and bioburden assessed. The gross pathology, of representative SCID mice that received splenocytes from singly infected (a) or doubly infected (b) BALB/c mice, following skin challenge is shown. Magnified images of skin lesion (marked with a circle) in some representative mice from various groups are also shown (c). (TIFF) [file ppat.1006122.s005.tiff]
